# Perinatal predictors of parenting stress trajectories during the first 3 years of parenthood: A Maternal and Child Health Handbook cohort study

**DOI:** 10.1002/jcv2.70154

**Published:** 2026-08-25

**Authors:** Nao Oikawa, Mariko Hosozawa, Syudo Yamasaki, Shuntaro Ando, Mitsuhiro Miyashita, Kaori Baba, Junko Niimura, Naomi Nakajima, Satoshi Yamaguchi, Yuko Morimoto, Sho Kanata, Hiroyasu Iso, Mariko Hiraiwa‐Hasegawa, Kiyoto Kasai, Atsushi Nishida

**Affiliations:** ^1^ Department of Pediatrics and Adolescent Medicine Juntendo University Tokyo Japan; ^2^ Research Center for Social Science & Medicine Tokyo Metropolitan Institute of Medical Science Tokyo Japan; ^3^ Institute for Global Health Policy Research Bureau of Global Health Cooperation Japan Institute for Health Security Tokyo Japan; ^4^ Department of Neuropsychiatry Graduate School of Medicine The University of Tokyo Tokyo Japan; ^5^ Department of Psychology Ube Frontier University Yamaguchi Japan; ^6^ Department of Psychiatry Teikyo University School of Medicine Tokyo Japan; ^7^ Department of Evolutionary Studies of Biosystems The Graduate University for the Advanced Studies, SOKENDAI Kanagawa Japan

**Keywords:** child, latent class analysis, longitudinal studies, parity, premature birth

## Abstract

**Background:**

Evidence of how parenting stress evolves during early childhood is limited. We aimed to identify parenting stress trajectories involving children aged 1 month to 3 years, and to determine their perinatal predictors.

**Methods:**

Data were obtained from 3133 mothers in the Tokyo Teen Cohort (TTC), a population‐based cohort of children born between 2002 and 2004. Parenting stress was self‐recorded in the Maternal and Child Health Handbook (MCHH) at eight time points between 1 and 36 months postpartum. Latent class growth analysis identified trajectories; multivariate multinomial logistic regression analyzed baseline predictors from the MCHH or the TTC first wave.

**Results:**

Five distinct trajectories were identified: consistently low (*n* = 1,691, 54.0%), consistently high (*n* = 158, 5.0%), consistently moderately high (*n* = 719, 22.9%), high‐decreasing (*n* = 299, 9.5%), and low‐increasing (*n* = 266, 8.5%). Compared with the consistently low group, primiparity predicted all trajectories with initially high stress: consistently high (adjusted odds ratio [aOR] 1.64, 95% confidence interval [CI] 1.13–2.37), consistently moderately high (aOR 1.92, 95% CI 1.57–2.35), and high‐decreasing (aOR 1.87, 95% CI 1.38–2.54). Preterm birth also predicted the consistently high trajectory (aOR 2.38, 95% CI 1.31–4.32). Maternal alcohol consumption during pregnancy (aOR 1.33, 95% CI 1.06–1.67) and male sex (aOR 0.80, 95% CI 0.66–0.97) predicted the consistently moderately high trajectory. High maternal education (aOR 1.58, 95% CI 0.99–2.52) tended to predict the low‐increasing trajectory. Maternal age, chronic health conditions, smoking during pregnancy, single‐parent household, and history of infertility treatment were not significant predictors.

**Conclusion:**

Parenting stress follows heterogenous trajectories, necessitating repeated assessments during the first 3 years. Integrating systematic, self‐recorded screenings into routine check‐ups could facilitate the timely identification of evolving stress patterns. Recognizing early predictors, including primiparity and preterm birth, can further assist in identifying mothers warranting closer longitudinal monitoring.

## Key Points

**What's known?**

- Little evidence exists on how parenting stress patterns develop during early childhood and their perinatal predictors in the general population.

**What's new?**

- This study identified five distinct parenting stress trajectories from 1 to 36 months postpartum: consistently low (54%), consistently high (5%), consistently moderately high (22.9%), low‐increasing (8.5%), and high‐decreasing (9.5%).
- Primiparity was universally associated with elevated stress at 1 month postpartum, whereas preterm birth predicted the consistently high stress trajectory.

**What's relevant?**

- The heterogeneous nature of parenting stress highlights the importance of repeated assessments across the first 3 years.
- Integrating self‐recorded tools such as the Maternal and Child Health Handbook into routine health check‐ups can be a scalable strategy to identify mothers who may benefit from further evaluation and support.

## INTRODUCTION

Parenting stress is defined as the aversive psychological and physiological reactions that arise when parenting demands exceed an individual’s resources (Deater‐Deckard, 2004). While parenting stress can occur at any stage of parenthood, it often peaks during the first few years as parents adjust to their new roles (Reijneveld et al., 2008). Over 50% of parents experience parenting concerns during early childhood (i.e., up to age 3), with stress levels declining thereafter (Reijneveld et al., 2008; Respler‐Herman et al., 2012). Heightened and prolonged parenting stress during this period is associated with negative parental health including psychological distress and maladaptive or less sensitive parenting behaviors (Deater‐Deckard, 2004; Respler‐Herman et al., 2012; Tedgard E, Tedgard U, Rastam, & Johansson, 2020), which may negatively affect parent‐child relationships and a child’s developmental outcomes, particularly behavioral and emotional outcomes (Senehi et al., 2025). Since early intervention can reduce parenting stress and its adverse effects on the family (Goodman et al., 2020), identifying perinatal risk factors for parenting stress is essential for detecting parents at high risk, particularly those susceptible to heightened and prolonged stress.

Parenting stress is influenced by various factors related to the parent, child, and family environment, and is likely to change as children develop and parenting demands evolve (Fang et al., 2024). However, most studies have assessed parenting stress at a single time point, and those that have conducted repeated assessments have primarily focused on average, population‐level trajectories. Person‐centered longitudinal approaches such as latent class growth analysis can identify heterogeneous trajectories rather than assuming a single average pattern of change within a population (Jung & Wickrama, 2008).

To date, studies examining parenting stress trajectory patterns are limited. Most existing studies have focused on specific high‐risk populations, such as young and socioeconomically disadvantaged parents (Chang & Fine, 2007; Huang et al., 2019), or parents of children with special healthcare needs (Golfenshtein et al., 2019). One exception is a study by Mulsow et al. (2002), which identified four parenting stress trajectory groups during the first 3 years of parenthood. However, that study included 134 mothers from a single site, with the largest group (approximately 40%) having experienced “chronically high” parenting stress. The relatively small and potentially non‐representative samples may have lacked sufficient statistical power to precisely identify groups, thereby limiting the generalizability of the findings.

Furthermore, identifying sociodemographic and perinatal predictors of parenting stress trajectories can inform clinical practice and public health policy. Higher parenting stress has been associated with primiparity (Fang et al., 2024), preterm birth (Gray et al., 2018; Spinelli et al., 2013), and maternal socioeconomic status, including lower educational attainment and single‐parent family structures (Nomaguchi & Brown, 2011; Putnick et al., 2020). Child factors, such as child sex, have also been associated with higher parenting stress (Dayton et al., 2017). Nevertheless, how these baseline factors predict distinct parenting stress trajectories across early childhood remains largely unexplored.

All expecting mothers in Japan receive a Maternal and Child Health Handbook (MCHH) from their local government under the Law of Maternal and Child Health. Healthcare providers review these responses as a screening tool during maternal or child health check‐ups. The MCHH contains questions concerning the mother’s health, family structure, and work status, which mothers are required to answer during pregnancy. It also contains sections for healthcare providers at birth facilities to record delivery details. After birth, mothers are asked to record their responses to questions on child development and parenting stress prior to each routine child health check‐up at 1, 3, 6, 9, 12, 18, 24, and 36 months. Globally, the MCHH flamework has been introduced in over 50 countries, including many Asian and African countries, to support maternal and child healthcare systems (Japan International Cooperation Agency, 2025).

In this study, we aimed to use parenting stress information recorded in the MCHH obtained from a large population‐based cohort in Tokyo, Japan, to identify parenting stress trajectories from 1 to 36 months postpartum, and their sociodemographic and perinatal predictors. We hypothesized that parenting stress trajectories during this period would be heterogeneous, including a consistently high parenting stress group. Based on previous literature, we further hypothesized that primiparity and preterm birth, would predict trajectories characterized by elevated parenting stress. Identifying parenting stress patterns and their predictors can inform the development of tailored and timely interventions and public health policies to better support those parents in need of additional support.

## METHODS

### Participants

This study utilized data from the Tokyo Teen Cohort (TTC), an ongoing population‐based longitudinal study investigating the health and developmental trajectories of children living in the Tokyo metropolitan area. The cohort comprises children born between September 2002 and August 2004, randomly selected based on resident registry data (Ando et al., 2019). Initial data collection was conducted between 2012 and 2014 when the children were 10 years old. Trained interviewers conducted surveys during in‐home visits, and transcribed data historically recorded in the MCHH. The MCHH is a universally distributed personal health record used in Japan since 1948 to track the health of the expecting mother and the child from pregnancy through childhood. Approximately 95% of pregnant women obtain their MCHH by 11 weeks of gestation, and 99% by 19 weeks of gestation (Ministry of Health Law 2025). Explicit data indicating the specific family member who completed the MCHH are unavailable; however, given the MCHH is structurally designated for maternal record‐keeping, it can be reasonably assumed that the respondents were the mothers.

Of the 3171 mothers of the enrolled children, 38 were excluded owing to missing parenting stress data at all 8 time points. The final sample for this study included 3133 (98.8%) mothers. A detailed description of the cohort has been reported elsewhere (Ando, Nishida, Yamasaki, et al., 2019). The study protocol was approved by the Ethics Committees of the Tokyo Metropolitan Institute of Medical Science (No. 12–35), the University of Tokyo (No. 10057), and the Graduate University for Advanced Studies, SOKENDAI (No. 2012002). Prior to the study, written informed consent was obtained from the parents, and informed assent was obtained from the children enrolled.

### Measurements

#### Parenting stress

Parenting stress was measured using the parents’ self‐reported responses recorded in the MCHH to the following question: “Do you feel parenting is difficult?” Possible responses were “yes” (coded as 2), “not sure” (coded as 1), and “no” (coded as 0). The question was self‐recorded at eight time points (i.e., 1, 3, 6, 9, 12, 18, 24, and 36 months postpartum) before the scheduled child health check‐ups. This question in the MCHH has previously been used to indicate parenting stress (Endo, Stanyon, Yamasaki, et al., 2022; Niimura, Nakanishi, Yamasaki, et al., 2022).

#### Predictors

The predictor variables were obtained either from the MCHH or from parental surveys at the first wave of the TTC when the children were aged 10 years. Variables derived from the MCHH included: preterm birth (defined as born before 37 weeks gestation or not based on a healthcare facility report); primiparity (primipara or not); maternal chronic health conditions (had been diagnosed with hypertension, chronic renal disease, diabetes mellitus, hepatitis, heart disease, a thyroid disorder, a psychiatric disorder, other specified conditions, or not); maternal alcohol consumption during pregnancy (yes or no); and cigarette smoking during pregnancy (yes or no), as reported by the mother. Maternal chronic health conditions, alcohol consumption, and cigarette smoking were reported in the first or second trimester. Variables obtained from the parent questionnaire at child age 10 included a history of infertility treatment for the child (yes or no), the child’s sex (male or female), maternal age at childbirth, the highest level of maternal education (having a high school degree or higher or lower), and whether the child was born into a single‐parent household (yes or no).

### Statistical analyses

We first conducted descriptive analyses to examine the distributions of the study’s variables. Next, a latent class growth analysis of parenting stress using a three‐step approach for latent class and growth mixture modeling was conducted to identify parenting stress trajectories (Asparouhov & Muthén, 2014). Missing data on the parenting stress measure at each time point ranged from 2.8% at 3 months to 17.3% at 1 month (Supporting Information S1: Table S1). Here, parenting stress was treated as a three‐level ordered categorical variable (i.e., yes, not sure, no), and missing data were treated using full‐information maximum likelihood estimation as multiple imputation was computationally intensive and less feasible for eight time points of an ordered categorical variable. We investigated two to six class models with linear and quadratic slopes, and the best‐fitting model was identified based on the Akaike information criterion, Bayesian information criterion, the Vuong–Lo–Mendell–Rubin likelihood ratio test, and the clinical meaning and proportion of the classes. Once the optimal model was identified, the association between latent trajectories and potential predictors was investigated in a multivariate multinomial logistic regression model, adjusting for classification error introduced with profile assignment, using the R3STEP command in Mplus Version 8.7 (Muthén & Muthén) software. All predictors were mutually adjusted in the model. The predictors were primiparity, preterm birth, maternal health condition, maternal cigarette smoking, maternal alcohol consumption, infertility treatment history, maternal age at childbirth, child sex, maternal education, and single‐parent household at birth. Missing predictor variables ranged from 0.5% for maternal age at childbirth to 12.8% for maternal alcohol consumption. We imputed missing predictor values to minimize bias owing to listwise deletion before conducting the multivariate multinomial logistic regression. These missing values were imputed using Bayesian estimation with 20 imputed datasets using all indicators and predictors in the model, and pooled using Rubin’s rules (White et al., 2011). The imputed results were similar to those using observed data (Supporting Information S1: Table S1). We did not adjust for multiple testing for our multivariable models as this was an exploratory study rather than a confirmative study. All analyses were conducted using Mplus 8.7 software.

## RESULTS

Among the 3113 parents included in the analysis, the mean maternal age at childbirth was 31.6 (standard deviation, 4.2) years. In total, there were 1152 (38.1%) primipara mothers; 161 (5.1%) children were born preterm, and 1660 (53.0%) children were boys. During pregnancy, 145 (5.2%) mothers actively smoked, while 741 (27.1%) consumed alcohol, and 2592 (83.3%) mothers had attained high maternal education (Table 1).

**TABLE 1:** Sociodemographic and perinatal characteristics of the observed samples (*N* = 3133).

Characteristic	*n*	Mean/*n*	%/(SD)
Maternal age at childbirth, mean (years)	3133	31.6	(4.2)
Primipara	3133	1152	38.1
Child born preterm (<37 weeks)	3133	161	5.1
Sex of the child, boy	3133	1660	53.0
Maternal smoking (yes)	2767	145	5.2
Maternal alcohol consumption (yes)	2731	741	27.1
Maternal chronic health condition (yes)	3133	197	6.3
Have taken infertility treatment (yes)	2981	323	10.8
High maternal education (above high school)	3110	2592	83.3
Single‐parent household (yes)	3119	64	2.1

*Note: n* varies due to missing data.

Abbreviations: *n*, number; SD, standard deviation.

Overall, parenting stress was highest at 1 month postpartum (“yes,” 12.8%; “not sure,” 28.9%), which decreased to 8.7% and 23.3%, respectively, by 3 months postpartum, and remained relatively stable until 36 months postpartum (Table 2).

**TABLE 2:** Proportion of parenting stress response category across different time points (*N* = 3133).

Time points	Parenting stress response			
	No	Not sure	Yes	Missing
	*n* (%)	*n* (%)	*n* (%)	*n* (%)
1 month	1511 (58.3)	748 (28.9)	333 (12.8)	541 (17.3)
3 months	2070 (68.0)	710 (23.3)	265 (8.7)	88 (2.8)
6 months	1909 (69.3)	607 (22.0)	238 (8.6)	379 (12.1)
9 months	2199 (72.8)	611 (20.2)	212 (7.0)	111 (3.5)
12 months	2003 (72.2)	564 (20.3)	209 (7.5)	357 (11.4)
18 months	2107 (69.5)	660 (21.8)	263 (8.7)	103 (3.3)
24 months	1932 (69.7)	594 (21.4)	247 (8.9)	360 (11.5)
36 months	2081 (68.6)	689 (22.7)	262 (8.6)	101 (3.2)

**FIGURE 1:**
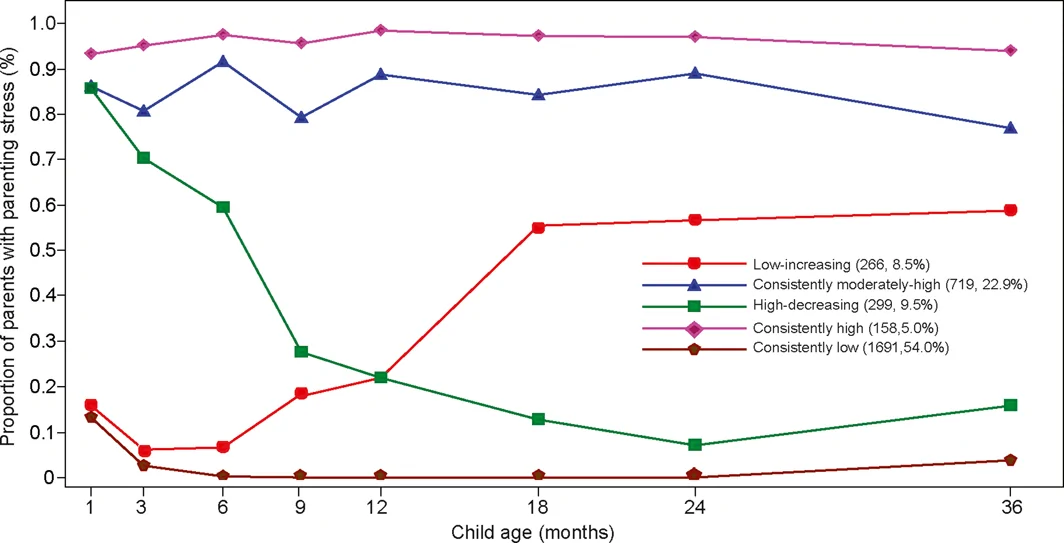
Proportion of parenting stress according to identified trajectories. Parenting stress trajectories across eight time points (age: 0–36 months) are shown.

Latent class growth analysis and model fitting indicated that the best‐fitting model was the five‐class model (Supporting Information S1: Table S2). Supporting Information S1: Table S3 presents the proportion of responses in each parenting stress category according to identified trajectories. In this five‐class model, the largest group of participants (*n* = 1691, 54.0%) experienced minimal or no parenting stress throughout the study (“consistently low” group). This was followed by a group who experienced moderate stress throughout the study period (*n* = 719, 22.9% of the “consistently moderate high” group). The group with the lowest number was the “consistently high” group (*n* = 158, 5.0%), which experienced consistently high parenting stress throughout the study period. Furthermore, a “high‐decreasing” group (*n* = 299, 9.5%) experienced high parenting stress one month postpartum; however, their parenting stress showed a decreasing trend thereafter until the child reached the age of 24 months. The “low‐increasing” group (*n* = 266, 8.5%) experienced low parenting stress for the first 6 months; however, their stress increased until 18 months and remained moderately high until 36 months (Figure 1).

Multivariate multinominal logistic regression analysis indicated that, compared with the consistently low trajectory, primiparity (adjusted odds ratio [aOR] 1.64, 95% confidence interval [CI] 1.13–2.37) and preterm birth (aOR 2.38, 95% CI 1.31–4.32) predicted the consistently high trajectory. Primiparity also predicted consistently moderately high (aOR 1.92, 95% CI 1.57–2.35) and high‐decreasing (aOR 1.87, 95% CI 1.38–2.54) trajectories. Male child sex and maternal alcohol consumption predicted the consistently moderately high trajectory (aOR 0.80, 95% CI 0.66–0.97 and aOR 1.33, 95% CI 1.06–1.67, respectively). Maternal education beyond high school tended to be associated with the low‐increasing trajectory (aOR 1.58, 95% CI 0.99–2.52) (Table 3).

**TABLE 3:** Adjusted odds ratios for predictors of parenting stress trajectories.^a^

	Low‐increasing			High‐decreasing			Consistently moderately high			Consistently high		
	(*n* = 266, 8.5%)			(*n* = 299, 9.5%)			(*n* = 719, 22.9%)			(*n* = 158, 5.0%)		
	aOR	95% CI		aOR	95% CI		aOR	95% CI		aOR	95% CI	
Maternal age at childbirth	1.01	0.97	1.04	0.98	0.95	1.02	1.00	0.98	1.02	1.01	0.96	1.06
Primipara	1.25	0.90	1.74	**1.87**	**1.38**	**2.54**	**1.92**	**1.57**	**2.35**	**1.64**	**1.13**	**2.37**
Child born preterm	0.62	0.26	1.49	1.13	0.60	2.15	1.15	0.75	1.78	**2.38**	**1.31**	**4.32**
Sex of the child (reference = girl)	0.81	0.60	1.10	0.84	0.62	1.14	**0.80**	**0.66**	**0.97**	1.13	0.79	1.61
Maternal smoking	0.75	0.33	1.70	0.48	0.18	1.28	0.71	0.44	1.16	1.27	0.60	2.68
Maternal alcohol consumption	0.85	0.57	1.27	1.04	0.73	1.50	**1.33**	**1.06**	**1.67**	1.15	0.75	1.77
Maternal chronic health condition	1.51	0.85	2.66	1.18	0.66	2.13	1.17	0.78	1.73	1.62	0.86	3.06
Have taken infertility treatment	1.21	0.75	1.93	1.44	0.91	2.28	1.09	0.78	1.52	0.83	0.44	1.56
High maternal education^b^	1.58	0.99	2.52	1.03	0.69	1.53	1.05	0.81	1.36	1.54	0.90	2.63
Single‐parent household	1.66	0.54	5.09	1.84	0.68	5.01	1.60	0.80	3.21	1.58	0.48	5.19

*Note:* All predictive variables were mutually adjusted in the model. The significance level was set at *p* < 0.05. Statistical significance is shown in bold.

Abbreviations: aOR, adjusted odds ratio; CI, confidence interval.

^a^ The “consistently low” group (*n* = 1691, 54.0%) serves as a reference.

^b^ Defined as having a high school degree or higher.

## DISCUSSION

Using MCHH data, five distinct parenting stress trajectories were identified during the first 3 years after birth in this population‐based cohort. While 54.0% of parents experienced minimal or no parenting stress throughout early childhood, 22.9% consistently experienced moderate parenting stress, and 5.0% experienced high parenting stress. Some parents (8.5%) experienced increasing parenting stress after 6 months postpartum, whereas others (9.5%) experienced decreasing stress as their child aged. Among the studied perinatal predictors, primipara and preterm birth were key predictors associated with the consistently high‐stress trajectories.

Despite the clinical and public health importance of understanding patterns of parenting stress and its predictors, few studies have examined parenting stress trajectories in the general population across early childhood. Our findings, which identified multiple parenting stress trajectories—including groups with consistently high and low parenting stress, as well as increasing and decreasing patterns, largely align with those of a United States study (Mulsow et al., 2002). However, notable differences in group distribution emerged. Muslow et al. reported that their largest group (40%) experienced chronically high parenting stress, followed by a chronically low parenting stress group (34%). In contrast, the largest group in our study had consistently low stress (53.9%), while the consistently high‐stress group was the smallest (5.0%). This distributional difference may be because our larger sample allowed us to identify the moderately high parenting stress group (22.9%), which may have been merged into the chronically high group in the previous study. Our study thus extends the previous findings by more precisely identifying this small but high‐risk group of parents who require intensive support. Systemic differences in maternal and child health systems between Japan and the United States may also explain the divergent findings. In Japan, municipalities provide universal child health check‐ups and/or structured self‐recording in the MCHH throughout infancy up to the third year of a child's life, including at approximately 1, 3–4, 6–7, 9–10, 12, 18, 24, and 36 months. This structured, continuous screening infrastructure may facilitate earlier identification and support parents experiencing parenting stress. This could have resulted in a greater proportion of mothers experiencing consistently low parenting stress in our study as compared to Mulsow et al.’s study (53.9% vs. 34.0%, respectively).

Primiparity predicted all trajectories with heightened stress at 1 month postpartum, even after adjusting for other potential predictors. First‐time parents often face difficulties managing childcare and adapting to their new roles, which can increase parenting stress, particularly during the early postpartum period (Fang et al., 2024). This increased stress can impair their relationships with the child or the family and may increase the risk of child maltreatment if left unsupported (McLearn et al., 2006; Niimura et al., 2022). One review highlighted the need for first‐time parents to receive early, realistic information about parenting, opportunities for peer learning, and access to support from health professionals when difficulties arise (Entsieh & Hallstrom, 2016). Our findings suggest that enhanced proactive support for first‐time parents should ideally begin during the antenatal period. Effective parental intervention may include structured education on child development and core parenting skills, and tailored routine assessments of parental needs. Because our study focused on baseline predictors, we were unable to consider the dynamic role of social support, such as support from partners, which is known to be crucial for coping with parenting stress (Fierloos, Windhorst, Fang, et al., 2023). The potential protective role of social support, including partner and community support, on parental stress trajectories should be explored in future research.

Approximately 5% of parents experienced consistently high parenting stress throughout early childhood, which was predicted by both primiparity and preterm birth. Preterm birth is often accompanied by periods of maternal‐infant separation and uncertainty regarding the child’s health and development, which can cause significant parental distress (Gray et al., 2018; Spinelli et al., 2013). Parents of preterm‐born children are reported to experience more long‐term psychological distress than parents of term‐born children, which can exacerbate parenting stress (Treyvaud et al., 2014). Our results highlight the need for continuous support for parents of preterm‐born children, from pregnancy through early childhood.

The second largest group in our study was the consistently moderately high trajectory, which was predicted by primiparity, maternal alcohol consumption during pregnancy, and child male sex. Male children are more likely to display externalizing behaviors during early childhood than females (Dayton & Malone, 2017), which may increase parenting stress. Furthermore, a previous study reported that higher levels of parenting stress from 9 months to 3 years of age were associated with later attention‐deficit/hyperactivity disorder diagnoses (Endo et al., 2022), which parental alcohol consumption during pregnancy is a known risk factor (Sciberras et al., 2017). Therefore, the persistent moderate parenting stress experienced in this group may partly relate to the child’s underlying characteristics, including neurodevelopmental characteristics (Nomaguchi & Brown, 2011). Alternatively, maternal alcohol consumption during pregnancy may reflect the mother's vulnerability to psychological distress or the use of alcohol as a coping strategy in response to stress. The findings suggest the need to assess signs of parenting distress for mothers who report alcohol consumption during pregnancy and the importance of conducting child development assessments, including neurodevelopmental characteristics, when supporting parents experiencing moderately high parenting stress for a prolonged period. Furthermore, future research incorporating detailed neurodevelopmental assessments may help clarify mechanisms linking child characteristics and parenting stress trajectories.

Our study also identified a group of parents who initially had low parenting stress, which increased as the child aged. This group, consisting of 8.5% of the total sample, was predicted by higher maternal education. This contrasts with previous studies reporting higher parenting stress among mothers with lower education levels (Putnick et al., 2020; Vispoel et al., 2023). However, highly educated parents may face greater challenges adjusting to their new parental role because of greater career investments (Parkes et al., 2015). Mothers with advanced degrees are more likely to be employed full‐time and may experience increased pressure to balance childcare and career demands, particularly as the child becomes more mobile after late infancy (Oddi et al., 2013). The finding suggests healthcare providers should acknowledge that parenting stress can increase after the first 6 months for some mothers, including highly educated mothers who might not have been previously identified as a high‐risk group. This underscores the necessity of repeated parenting stress assessments. Utilizing self‐recorded MCHH and leveraging routine health check‐ups, as in our study, can provide an opportunity for early identification of increased parenting stress and implementation of preventive support strategies among mothers in the general population. Furthermore, a previous study on maternal depression during the first 3 years reported that 8.2% of mothers initially exhibited low depressive symptoms that increased over time (Putnick et al., 2020), which may contribute to the observed later increase in parenting stress in this group. Future studies investigating the role of maternal depression on parenting stress trajectories could provide more insight into the causes of parenting stress during early childhood and potential intervention strategies.

The strengths of this study included its population‐based design with a high inclusion rate (99% of the original sample), a large sample size compared with other studies on parenting stress, and the use of parenting stress data transcribed from the prospectively recorded MCHH, minimizing recall bias.

However, our study had some limitations. The observational design of the study precludes drawing any causal relationships. Parental stress was assessed based on self‐reports, which may be subject to social desirability bias (Vispoel et al., 2023). The use of a single item to assess parenting difficulties may not fully capture the multidimensional nature of parenting stress. Nevertheless, subjective perception of parenting difficulties is a core component of parenting stress theory (Deater‐Deckard, 2004), supporting the utility of this parenting stress measure recorded in the MCHH as a screening indicator to identify mothers experiencing parenting stress in real‐world settings. The study’s predictors were limited primarily to variables available in the MCHH at childbirth to inform clinical and public health policy. Consequently, we could not consider factors that emerge during the course, such as changes in family structure or the family’s socioeconomic status, which might have influenced the trajectories. Relatedly, maternal education was measured when a child was age 10. However, because educational attainment in Japan is typically completed during early adulthood, and subsequent changes after childbirth are rare, it possibly serves as a reliable proxy for socioeconomic status during early childhood. Furthermore, information on postpartum depression was not available in the MCHH at the time of this study. However, the most recent version of the MCHH includes a question concerning postpartum depression at 1 month, which can be explored in future studies. Finally, our study tracked parenting stress until the child was 3 years old; examining it beyond this period is important for designing effective long‐term interventions for those who need them. Nevertheless, early childhood is a crucial period when parenting stress is at its peak, highlighting the importance of investigating this critical stage.

## CONCLUSION

Maternal and child healthcare providers should acknowledge that parenting stress across early childhood follows heterogenous trajectories, highlighting the necessity of repeated assessments to identify varying parental needs. Early identification of mothers experiencing persistently high parenting stress, particularly first‐time mothers and mothers of preterm children, can allow healthcare providers to offer closer monitoring. Furthermore, given that some parents experience increased parenting stress well after the initial postpartum period, assessments of parenting stress should extend throughout early childhood. Leveraging routine child health check‐ups to track these trajectories using the self‐recorded parenting stress item in the MCHH offers a practical and scalable screening strategy. Ultimately, implementing these systematic assessments provides a framework to identify at‐risk mothers, facilitating timely intervention to promote parental well‐being, and foster optimal child development.

## AUTHOR CONTRIBUTIONS

**Nao Oikawa**: Conceptualization; study design; formal analysis; writing—original draft preparation. **Mariko Hosozawa**: Conceptualization; study design; formal analysis; writing—original draft preparation. **Syudo Yamasaki**: Conceptualization; study design; writing—review and editing. **Shuntaro Ando**: Survey designing; data collection; writing—review and editing. **Mitsuhiro Miyashita**: Survey designing; data collection; writing—review and editing. **Kaori Baba**: Survey designing; data collection; writing—review and editing. **Junko Niimura**: Survey designing; data collection; writing—review and editing. **Naomi Nakajima**: Survey designing; data collection; writing—review and editing. **Satoshi Yamaguchi**: Survey designing; data collection; writing—review and editing. **Yuko Morimoto**: Survey designing; data collection; writing—review and editing. **Sho Kanata**: Survey designing; data collection; writing—review and editing. **Hiroyasu Iso**: Supervision; writing—review and editing. **Mariko Hiraiwa‐Hasegawa**: Supervision; writing—review and editing. **Kiyoto Kasai**: Supervision; writing—review and editing. **Atsushi Nishida**: Conceptualization; study design; writing—review and editing.

## CONFLICT OF INTEREST STATEMENT

The authors declare no conflicts of interest.

## ETHICAL CONSIDERATIONS

This study was formally approved by the ethics committees of three sponsoring institutions on August 6, 2012, namely, the Tokyo Metropolitan Institute of Medical Science (approval number: 12–35), the University of Tokyo (approval number: 10057), and the Graduate University for Advanced Studies (approval number: 2012002). Prior to the study, written informed consent was obtained from the parents, and informed assent was obtained from the children enrolled.

## Supporting information

Supporting Information S1

## Data Availability

Deidentified data can be shared based on reasonable requests to the corresponding author and approval of the steering committee.
