# Supplementary material for: Perinatal predictors of parenting stress trajectories during the first 3 years of parenthood: A Maternal and Child Health Handbook cohort study
Source: JCPP Adv. 2026 Aug 25:e70154. Online ahead of print. doi: 10.1002/jcv2.70154 (PMC13507685; doi:10.1002/jcv2.70154)
Supplement: Supplementary file 1 [file JCV2-9999-e70154-s001.docx]

**Perinatal predictors of parenting stress trajectories during the first three years of parenthood:**

**a Maternal and Child Health Handbook cohort study**

# Supporting Information

# Table of Content:

# Table S1. Descriptive characteristics of the imputed sample (N = 3,133)

# Table S2. Model fit criteria for 2–5 class models of parenting stress trajectories (N = 3,133)

# Table S3. Proportion of responses in each parenting stress category according to identified trajectories

# Table S1. Descriptive characteristics of the imputed sample (N = 3,133)

|  | Mean / % | 95% CI |
| --- | --- | --- |
| Maternal age at childbirth, mean (years) | 31.6 | 31.5–31.8 |
| Primipara |  |  |
| No | 61.7 | 60.2–63.9 |
| Yes | 38.3 | 36.1–39.8 |
| Child born preterm (<37 weeks gestational age) |  |  |
| No | 94.7 | 94.2–95.8 |
| Yes | 5.3 | 4.2–5.8 |
| Child sex |  |  |
| Boy | 53.1 | 51.3–54.8 |
| Girl | 46.9 | 45.2–48.7 |
| Maternal smoking |  |  |
| No | 94.7 | 93.8–95.5 |
| Yes | 5.3 | 4.5–6.2 |
| Maternal alcohol consumption |  |  |
| No | 72.9 | 71.3–74.9 |
| Yes | 27.1 | 25.1–28.7 |
| Maternal chronic health condition |  |  |
| No | 93.8 | 93.0–94.6 |
| Yes | 6.2 | 5.4–7.0 |
| Have taken infertility treatment |  |  |
| No | 89.2 | 88.1–90.2 |
| Yes | 10.8 | 9.8–11.9 |
| Maternal education |  |  |
| High school or below | 16.5 | 82.2–84.7 |
| Above high school | 83.5 | 15.3–17.8 |
| Single-parent household |  |  |
| No | 97.9 | 97.5–98.3 |
| Yes | 2.1 | 1.7–2.5 |

CI, confidence interval

# Table S2. Model fit criteria for 2–5 class models of parenting stress trajectories (N = 3,133)

| Class models | 2 | | 3 | | 4 | | 5 | | 6 | |
| --- | --- | --- | --- | --- | --- | --- | --- | --- | --- | --- |
| AIC | 27474.21 | | 25883.66 | | 25406.88 | | 24881.54 | | 24774.56 | |
| Adjusted BIC | 27522.61 | | 25956.26 | | 25503.67 | | 24938.99 | | 24843.50 | |
| Entropy | 0.92 | | 0.93 | | 0.84 | | 0.87 | | 0.88 | |
| Vuong–Lo–Mendell–Rubin test | <0.0001 | | <0.0001 | | 0.0003 | | <0.0001 | | 0.125 | |
| Class membership | n | % | n | % | n | % | n | % | n | % |
| Class 1 | 2,021 | 64.5 | 1,954 | 62.4 | 1,669 | 53.3 | 1,691 | 54.0 | 1,673 | 54.0 |
| Class 2 | 1,112 | 35.5 | 994 | 31.8 | 756 | 24.1 | 719 | 22.9 | 714 | 22.8 |
| Class 3 |  |  | 184 | 5.9 | 553 | 17.7 | 299 | 9.5 | 321 | 10.2 |
| Class 4 |  |  |  |  | 155 | 4.9 | 266 | 8.5 | 237 | 7.6 |
| Class 5 |  |  |  |  |  |  | 158 | 5.0 | 160 | 5.4 |
| Class 6 |  |  |  |  |  |  |  |  | 28 | 0.9 |

AIC, Akaike information criteria; BIC, Bayesian information criteria

# Table S3. Proportion of responses in each parenting stress category according to identified trajectories

| Trajectory groups | Time points (postpartum) | Category 1 (‘No’) | Category 2 (‘Not sure’) | Category 3  (‘Yes’) |
| --- | --- | --- | --- | --- |
| Consistently low | 1 month | 86.5 | 12.8 | 0.7 |
|  | 3 months | 97.4 | 2.5 | 0.1 |
|  | 6 months | 99.7 | 0.3 | 0.0 |
|  | 9 months | 100.0 | 0.0 | 0.0 |
|  | 12 months | 100.0 | 0.0 | 0.0 |
|  | 18 months | 100.0 | 0.0 | 0.0 |
|  | 24 months | 100.0 | 0.0 | 0.0 |
|  | 36 months | 96.1 | 3.7 | 0.2 |
| High-decreasing | 1 month | 13.3 | 63.7 | 23.0 |
|  | 3 months | 23.9 | 63.4 | 12.7 |
|  | 6 months | 44.9 | 49.8 | 5.3 |
|  | 9 months | 64.8 | 32.7 | 2.4 |
|  | 12 months | 78.4 | 20.4 | 1.2 |
|  | 18 months | 90.2 | 9.3 | 0.5 |
|  | 24 months | 93.0 | 6.7 | 0.3 |
|  | 36 months | 83.6 | 15.5 | 0.9 |
| Low-increasing | 1 month | 94.3 | 5.4 | 0.3 |
|  | 3 months | 91.6 | 8.0 | 0.4 |
|  | 6 months | 86.0 | 13.3 | 0.7 |
|  | 9 months | 78.5 | 20.3 | 1.2 |
|  | 12 months | 69.8 | 28.2 | 1.9 |
|  | 18 months | 52.7 | 43.3 | 3.9 |
|  | 24 months | 40.6 | 53.1 | 6.3 |
|  | 36 months | 34.5 | 57.5 | 8.0 |
| Consistently  moderately high | 1 month | 17.7 | 64.8 | 17.5 |
|  | 3 months | 17.7 | 64.8 | 17.6 |
|  | 6 months | 17.7 | 64.8 | 17.5 |
|  | 9 months | 17.8 | 64.8 | 17.4 |
|  | 12 months | 18.0 | 64.8 | 17.3 |
|  | 18 months | 18.5 | 64.7 | 16.8 |
|  | 24 months | 19.3 | 64.6 | 16.1 |
|  | 36 months | 21.8 | 64.1 | 14.1 |
| Consistently high | 1 month | 1.1 | 19.0 | 79.9 |
|  | 3 months | 0.9 | 16.2 | 82.8 |
|  | 6 months | 0.7 | 13.2 | 86.1 |
|  | 9 months | 0.6 | 11.2 | 88.2 |
|  | 12 months | 0.5 | 10.0 | 89.5 |
|  | 18 months | 0.5 | 9.2 | 90.3 |
|  | 24 months | 0.6 | 10.5 | 89.0 |
|  | 36 months | 1.5 | 23.2 | 75.3 |
